# A30P mutant α-synuclein impairs autophagic flux by inactivating JNK signaling to enhance ZKSCAN3 activity in midbrain dopaminergic neurons

**DOI:** 10.1038/s41419-019-1364-0

**Published:** 2019-02-12

**Authors:** Zhinian Lei, Guangliang Cao, Gang Wei

**Affiliations:** Department of Human Anatomy, Medical School of Southeast University, Dingjiaqiao 87, Nanjing, Jiangsu 210009 P. R. China

## Abstract

Mutations in α-synuclein gene have been linked to familial early-onset Parkinson’s disease (PD) with Lewy body pathology. A30P mutant α-synuclein is believed to suppress autophagic progression associated with PD pathogenesis. However, the mechanistic link between A30P mutation and autophagy inhibition in PD remains poorly understood. In this study, we identified that A30P mutant α-synuclein resulted in reduced autophagy flux through promoting the decrease of autophagosomal membrane-associated protein LC3 and the increase of SQSTM1/p62 protein levels in midbrain dopaminergic neuron, due to the transcriptional repressor ZKSCAN3 trafficking from the cytoplasm to the nucleus. Moreover, the results demonstrated that A30P mutant α-synuclein not only decreased the phospho-c-Jun N-terminal Kinase (p-JNK) levels in midbrain dopaminergic neuron but also interfered autophagy without influencing the activities of AMPK and mTOR. Collectively, the present study reveals a novel autophagy inhibition mechanism induced by A30P mutant α-synuclein via transcriptional activation of the ZKSCAN3 in a JNK-dependent manner.

## Introduction

Parkinson’s disease (PD) is the second most common neurodegenerative disorder characterized by the progressive degeneration of dopaminergic (DA) neurons in the substantia nigra pars compacta of the midbrain as a result of the formation of intraneuronal inclusions called Lewy bodies and Lewy neurites, mainly composed of α-synuclein^1–3^. α-Synuclein is a 14-kDa, intrinsically disordered protein and mainly localizes in the presynaptic nerve terminals. Its physiological function remains undefined. Compelling lines of evidence shows that abnormally high amount of α-synuclein is a core player in the DA neuronal death in PD^4^. Aggregated and mutated α-synuclein results in perturbation of lysosomal function and causes significant function alterations and loss of Ca^2+^ homeostasis in mitochondria which in turn results in increased oxidative stress in PD brain^5^. Compared with wild-type α-synuclein, both in vitro and animal model studies indicate that A30P mutation greatly accelerates α-synuclein oligomerization^6^. Moreover, pathologically aggregated α-synuclein may spread from one neuron to the other throughout the brain during PD pathogenesis, widely implicated α-synuclein as a central part of the disease process^7,8^. These pathological changes could result in a cellular stress condition that interferes with intracellular clearance pathways and favor α-synuclein further aggregation. Therefore, clearance or degradation of misfolded and aggregated α-synuclein is a logical approach to effectively block the pathological progression of PD.

The ubiquitin–proteasome system (UPS) or autophagy–lysosome pathway (ALP) has been suggested to contribute to α-synuclein turnover. Although UPS can degrade both wild-type and mutant α-synuclein, wild-type α-synuclein is degraded mainly by chaperone-mediated autophagy (CMA) and the clearance of mutant α-synuclein is strongly dependent on macroautophagy, hereafter referred to as autophagy^9^. α-Synuclein overexpression inhibits autophagy by inhibition of GTPase Rab1a; this results in the mislocalization of Atg9, and defective autophagosome formation^10^. Aberrant α-synuclein also induces the autophagic mitochondrial removal (mitophagy)^11^. Moreover, A30P mutant α-synuclein impairs autophagic flux in rat primary midbrain neurons^12^. Patients carrying the A30P mutation demonstrate typical early-onset PD with mild form of dementia^13^. However, the mechanisms underlining α-synuclein degradation in DA neurons remains elusive.

Recent results have shown that transcription factors play an important role in the regulation of autophagy^14,15^. ZKSCAN3 (ZNF306), a zinc finger transcription factor harboring Krüppel-associated box (KRAB) and SCAN domains, resides on chromosome 6p22.1^16,17^. As a DNA-binding protein, it plays an important role in several cellular functions including maintenance of the nucleolus, neoplastic transformation, cellular proliferation, and apoptosis^18,19^. Cyclin D2, integrin β4, and vascular endothelial growth factor (VEGF) are its direct downstream gene targets^17,20^. EGFR (epidermal growth factor receptor), Cyclin D1, NF-κB, mitogen-activated protein kinase kinase 2 (MEK2), and insulin-like growth factor-2 (IGF-2) may represent ZKSCAN3 downstream targets as well. Furthermore, it antagonizes the transcription factor EB (TFEB)-mediated autophagy activity^21–23^. ZKSCAN3 represses transcription of more than 60 TFEB target genes involved in autophagy (including LC3, ULK1, and WIPI2) and lysosomal functions. Silencing ZKSCAN3 enhances autophagosome biogenesis and the mRNA level of several lysosomal genes. mTORC1 inhibition induces ZKSCAN3 accumulation in the cytosol. During starvation, protein kinase C activated c-Jun N-terminal Kinase (JNK) and p38 MAPK, then phosphorylated ZKSCAN3 and contributed to its nuclear export^21,24,25^. Therefore, therapeutically, the inhibition of ZKSCAN3 could be viewed as an attractive target to increase autophagy. In this study, we report that A30P mutant α-synuclein suppresses autophagy activity by decreasing the induction of JNK-mediated ZKSCAN3 nuclear translocation in midbrain dopaminergic neuron.

## Results

### Mutant A30P α-synuclein overexpression impairs autophagy

**Fig. 1:**
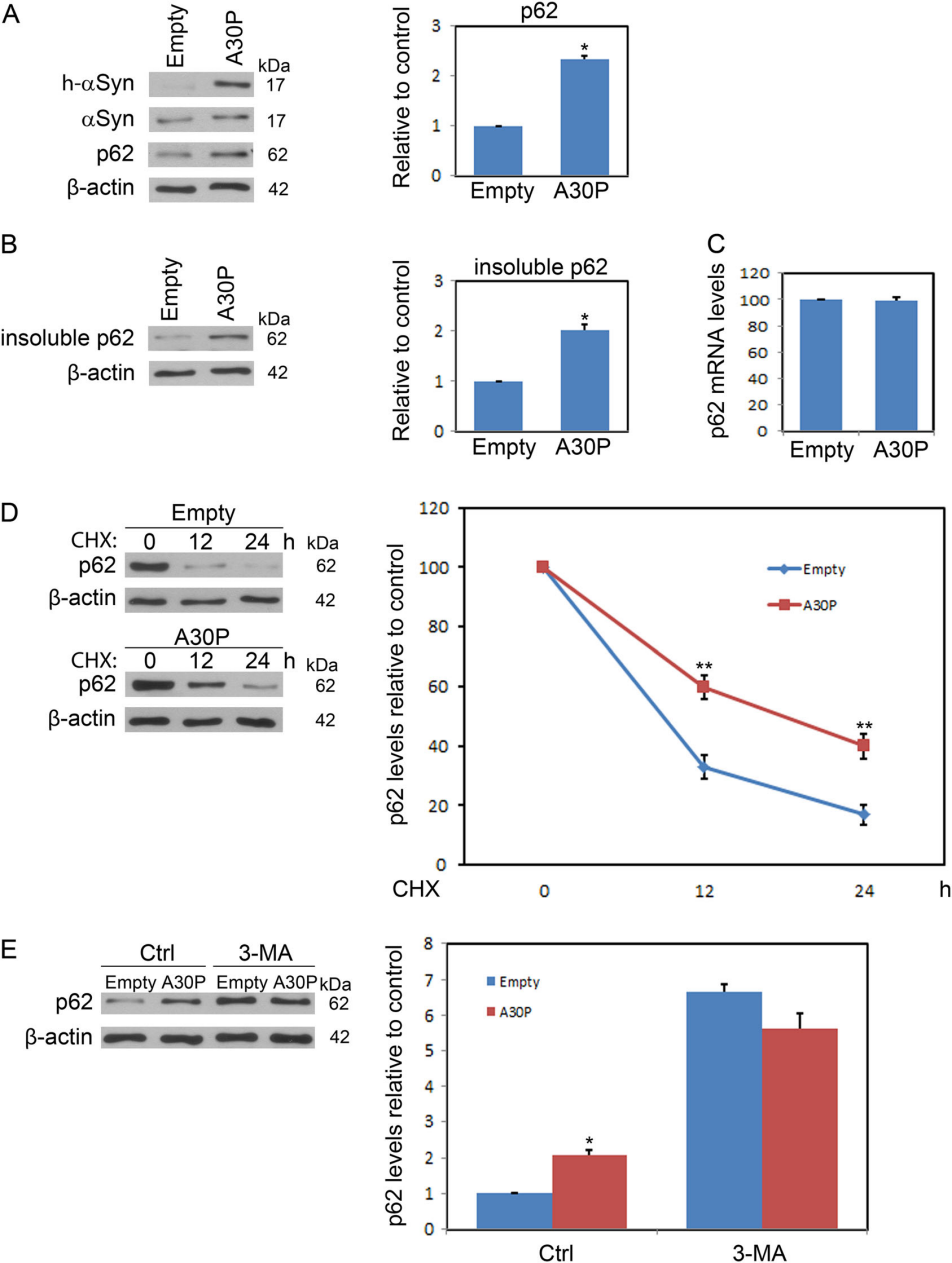
A30P α-synuclein overexpression hampers autophagy. **a** To investigate the effect of mutant A30P α-synuclein expression on autophagy, midbrain dopaminergic neurons expressing A30P α-synuclein were harvested and subjected to immunoblot analysis. P62 was used as an indicator for assessing autophagic activity. The intensity of each immunoblot band was quantified by densitometric scanning. **b** The insoluble fractions from midbrain dopaminergic neurons transfected with A30P α-synuclein were solubilized and separated by SDS-PAGE. **c** mRNA extracted from midbrain dopaminergic neurons transfected with A30P α-synuclein was used to assess amounts of p62 transcript relative to β-actin by RT-PCR. **d** A cycloheximide (10 μg/ml) chase experiment demonstrated that the decay kinetics of p62 in A30P α-synuclein-expressing cells was significantly prolonged compared with that in control group cells. The densitometric ratio is presented in the right panel. **e** midbrain dopaminergic neurons expressing A30P α-synuclein were treated or not with 3-MA (10 mM) for 24 h and then subjected to p62 immunoblotting. The results are presented as the means ± SEM from 3 independent experiments. The data were statistically analyzed by Student *t*-test or one-way ANOVA with Tukey’s post-hoc correction were used as indicated. *P < 0.05; **P < 0.01

To explore the effects of mutant A30P α-synuclein on autophagy, midbrain dopaminergic neurons were transfected with empty or human mutant A30P α-synuclein AAV vectors. The levels of SQSTM1/p62 (hereafter referred to as p62) were then determined by immunoblotting. As a LC3-adaptor protein, p62 is degraded by autophagy. Autophagy deficiency results in p62 aggregation and therefore p62 is a reliable reporter for evaluating autophagic activity. Representative immunoblots and quantitative cumulative data are shown in Fig. 1a. These data demonstrate that mutant A30P α-synuclein results in significant increases in p62 levels. To demonstrate that the increase in p62 levels after mutant A30P α-synuclein transfection was mediated by autophagy inhibition; the levels of insoluble p62 were determined by immunoblotting in midbrain dopaminergic neurons. These results clearly demonstrate that mutant A30P α-synuclein results in significant increases in insoluble p62 levels (Fig. 1b). In addition, the changes of p62 protein levels were not due to increased synthesis because mutant A30P α-synuclein had no effect on p62 messenger RNA levels (Fig. 1c). These data indicate that the accumulation of p62 may be resulted from inefficient clearance. Then, the degradation kinetics of p62 were examined by using cycloheximide, the endogenous p62 turnover was more slowly in mutant A30P α-synuclein-treated group (Fig. 1d). The p62 increases resulted from mutant A30P α-synuclein treatment may be an autophagy-dependent manner, we investigated the effects of mutant A30P α-synuclein on p62 protein levels under autophagy-deficient conditions. Midbrain dopaminergic neurons were treated with autophagy inhibitor 3-MA for 24 h, low-dose 3-MA increased the levels of p62. On the contrary, high-dose 3-MA did not further enhance the levels of p62 (Fig. S1 A), and impaired the cell viability of midbrain dopaminergic neuron (Fig. S1 B). When cells were treated with 10 mM 3-MA, the levels of p62 were increased in both the mutant A30P α-synuclein and empty vector group cells. However, no additional p62 increases were found in cells transfected with mutant A30P α-synuclein compared to that of control cells, which indicated the decreased autophagy flux (Fig. 1e). Our results suggest that p62 increases induced by A30P α-synuclein was due to dysfunctional autophagy.

### Autophagy inhibition induced by A30P α-synuclein correlates with ZKSCAN3

**Fig. 2:**
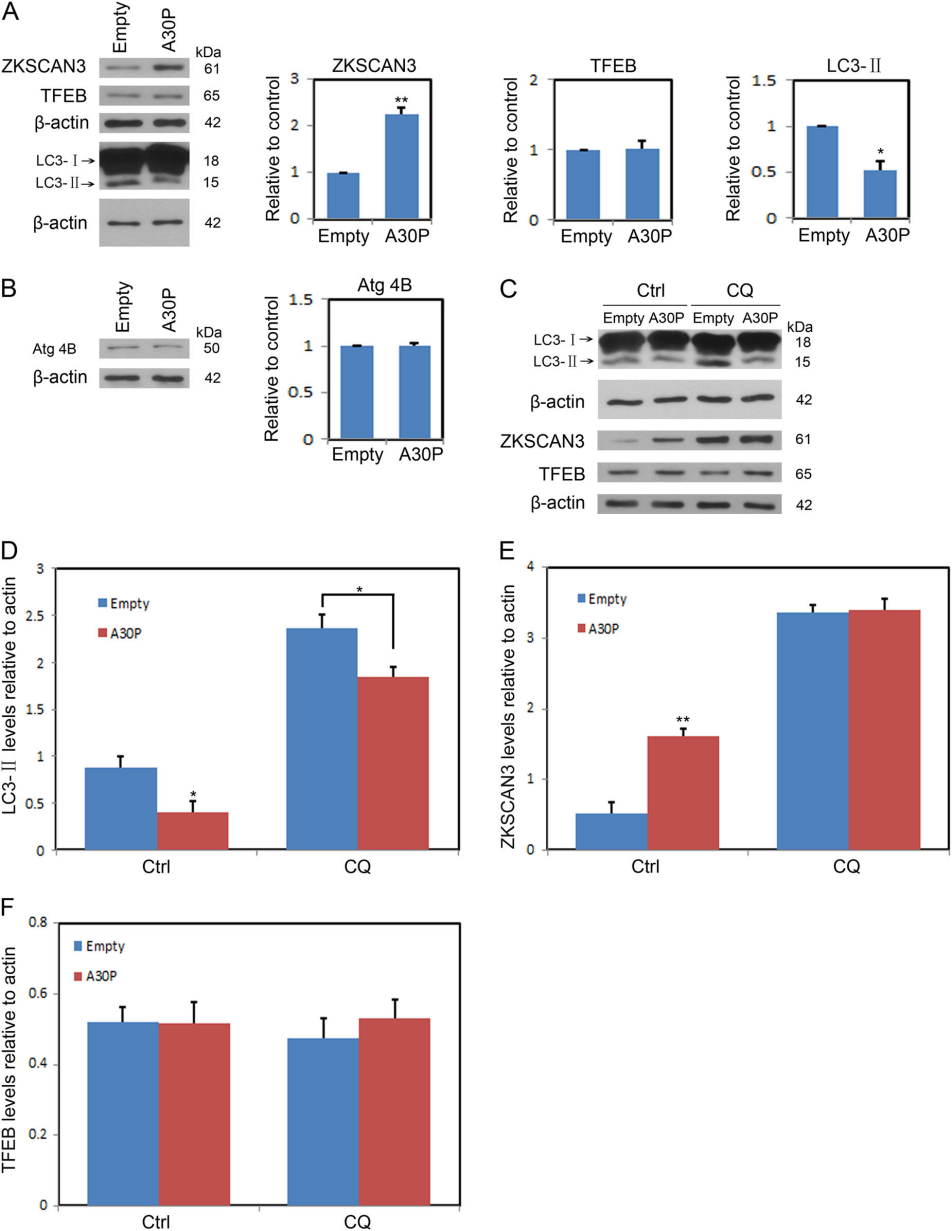
A30P α-synuclein overexpression inhibits autophagosome formation. **a** Representative ZKSCAN3, TFEB or LC3 immunoblots of whole cell protein lysates from midbrain dopaminergic neurons transfected with A30P α-synuclein for 48 h given above. Densitometric analyses were performed and shown in the right panel. **b** Midbrain dopaminergic neurons expressing A30P α-synuclein were lysted and immunoblotted with an anti-Atg 4B antibody. **c** Midbrain dopaminergic neurons expressing A30P α-synuclein were treated with and without CQ (10 μM) for 24 h and then lysted and subjected to SDS-PAGE. **d**–**f** Shown is the quantification of relative levels of ZKSCAN3, TFEB and LC3 remaining after treatment described in **c**. Data were shown as means ± SEM from three independent experiments. *P < 0.05; **P < 0.01 by Student *t-*test

To address the possible autophagic response influenced by A30P α-synuclein, midbrain dopaminergic neurons transfected with an empty vector or A30P α-synuclein before blotting for LC3-II as a marker of autophagosome formation. The levels LC3-II were robustly reduced in neurons expressing A30P α-synuclein compared to that in control cells (Fig. 2a). The transcriptional factor ZKSCAN3 or TFEB has been implicated in genes associated with autophagosome formation. A30P α-synuclein apparently increased the expression level of ZKSCAN3, but had no effect on that of TFEB (Fig. 2a). Upon autophagy initiation, LC3 is cleaved at the c-terminus by Atg4B to form cytosolic LC3-I, which is converted to LC3-II when conjugated to phosphatidylethanolamine. In our paradigms, A30P α-synuclein did not change the expression level of Atg4B, which suggested that there is no deficits in LC3 cleavage (Fig. 2b). The decreases in LC3-II levels may be resulted from either reduced synthesis or enhanced degradation by lysosome. To verify the two possibilities, neurons expressing A30P α-synuclein were incubated with vehicle only or lysosome-alkalizing agent chloroquine (CQ, 100 μM) for 12 h, to inhibit the acidification of lysosomes which decrease the activity of the acid hydrolases and block LC3-II degradation. The results indicated that the levels of LC3-II were still lower in neurons expressing A30P α-synuclein than that in control cells under treatment with CQ (Fig. 2c, d), showing reduced synthesis rather than enhanced degradation of LC3-II by A30P α-synuclein. Moreover, contrasted with TFEB, when treated with CQ, the levels of ZKSCAN3 were increased in neurons expressing A30P α-synuclein or empty vector. But no additional ZKSCAN3 increases were found in neurons expressing A30P α-synuclein compared to that of control cells (Fig. 2c, e and f). Our data convinced that A30P α-synuclein disrupted autophagy at the stage of autophagosome formation.

### Genetic manipulation of ZKSCAN3 rescues the autophagy inhibition effect induced by A30P α-synuclein

**Fig. 3:**
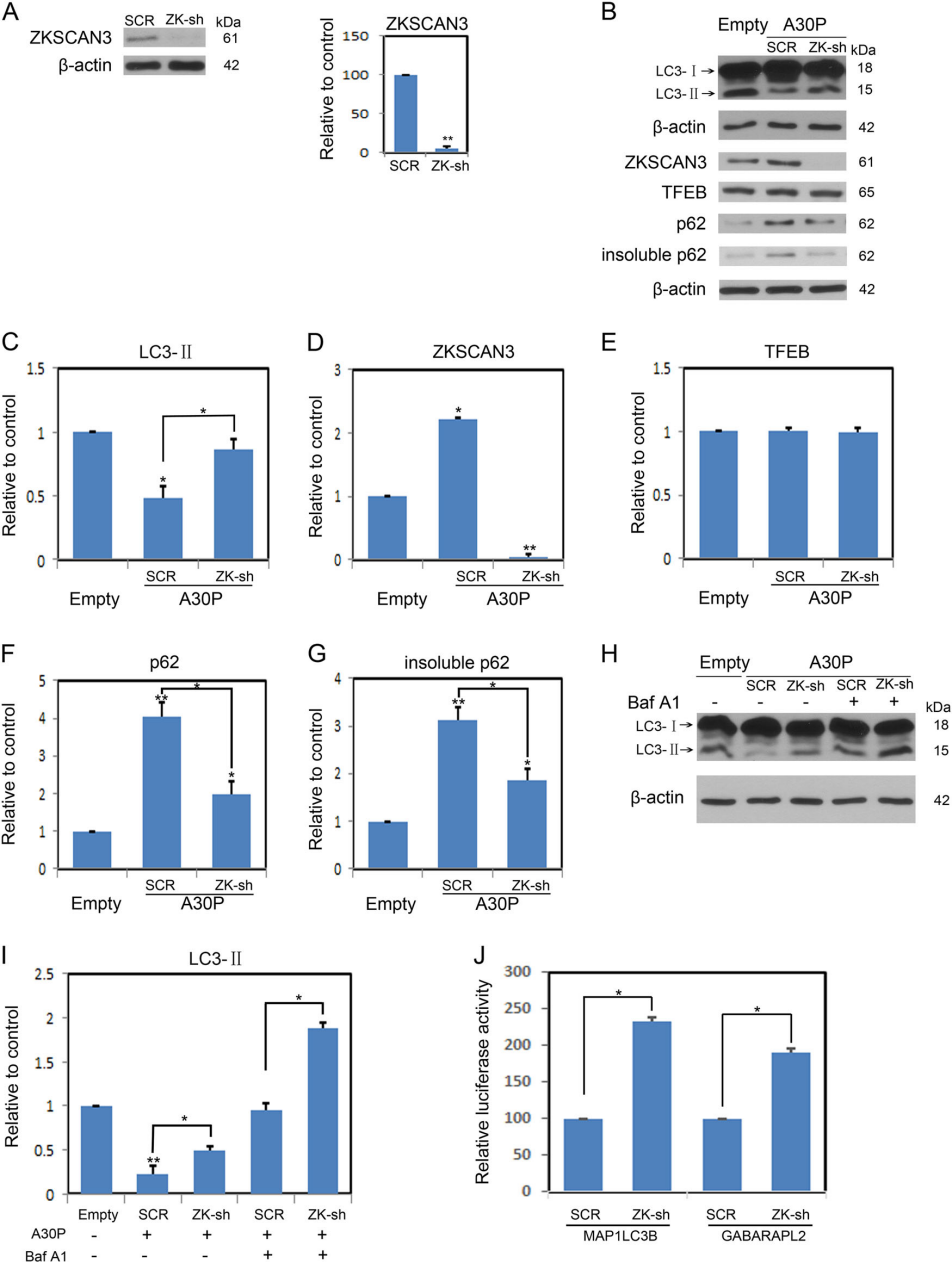
ZKSCAN3 is required for autophagy inhibition induced by A30P α-synuclein. **a** ZKSCAN3 shRNA apparently inhibited ZKSCAN3 levels. **b** Representative immunoblots of lysates from midbrain dopaminergic neurons expressing A30P α-synuclein were infected with either a scramble (SCR) or ZKSCAN3 shRNA virus. Total protein extracts were immunoblotted for LC3, ZKSCAN3, TFEB or p62. **c**–**g** Shown is the densitometric quantification of corresponding protein levels described in **b**. **h** Midbrain dopaminergic neurons expressing A30P α-synuclein were infected with either a SCR or ZKSCAN3 shRNA virus. Then, neurons were treated with and without Bafilomycin A1 (BafA1, 10 nM) for 12 h. LC3-I/II levels were analyzed by immunoblotting. **i** The histogram plot shows the densitometric quantification of LC3-II levels. **j** Luciferase reporter assay for *MAP1LC3B* or *GABARAPL2* constructs in midbrain dopaminergic neurons expressing A30P α-synuclein stably transfected either scramble (SCR) or ZKSCAN3-shRNA virus. Values are shown as mean ± SEM (*n* = 3). Immunoblots are representative of *n* = 3. *P < 0.05; **P < 0.01 by Student *t*-test. Abbreviations: shRNA, small hairpin RNA

To examine whether ZKSCAN3 is responsible for A30P α-synuclein-induced autophagy inhibition, midbrain dopaminergic neurons were transduced with a ZKSCAN3-shRNA or a control ZKSCAN3-scrRNA AAV vector. ZKSCAN3, TFEB, LC3-II and p62 proteins were then examined (Fig. 3a, b). ZKSCAN3-shRNA apparently inhibited the ZKSCAN3 levels (Fig. 3a, b and d). Knockdown of ZKSCAN3 significantly increased LC3-II levels in the neurons expressing A30P α-synuclein, whereas no TFEB changes were observed in the same treated neuron (Fig. 3b, c and e). Furthermore, ZKSCAN3-shRNA robustly repressed p62 protein aggregation in A30P α-synuclein-treated neurons (Fig. 3b, f and g) and rescued the decreased cell viability in midbrain dopaminergic neurons expressing A30P α-synuclein (Fig. S2 G). Although ZKSCAN3-shRNA significantly decreased the ZKSCAN3 levels in the neurons expressing WT α-synuclein (Fig. S2 A, B and D), knockdown of ZKSCAN3 had no apparent effect on levels of LC3-II and TFEB (Fig. S2 B, C and E). Moreover, ZKSCAN3-shRNA robustly reduced the p62 levels in WT α-synuclein-treated neurons (Fig. S2 B and F). Autophagy inhibition induced by A30P α-synuclein may be due to its delivery malfunction to lysosomes for degradation. As a vacuolar-type H^+^ ATPase inhibitor, Bafilomycin A1 (BafA1) prevents fusions of autophagosomes and lysosomes, thus inhibiting the autophagic flux. Midbrain dopaminergic neurons expressing A30P α-synuclein were transduced with ZKSCAN3-shRNA or a control ZKSCAN3-scrRNA. The results showed that BafA1 incubation increased the levels of LC3-II, and that in the presence of ZKSCAN3-shRNA there was a further increase (Fig. 3h, i), indicating that ZKSCAN3 repression does increase autophagy in neurons expressing A30P α-synuclein. The previous study has shown that ZKSCAN3 could directly regulate *MAP1LC3B* and *GABARAPL2* gene transcription^21^. *MAP1LC3B* and *GABARAPL2* promoter reporter constructs were transiently transfected into ZKSCAN3-shRNA and scramble neurons and dual luciferase assays were performed. The results revealed that promoter activity of both genes was apparently enhanced in ZKSCAN3-shRNA cells compared to control cells (Fig. 3j). These data indicate that knockdown of ZKSCAN3 specifically prevents the autophagy inhibition induced by A30P α-synuclein.

**Fig. 4:**
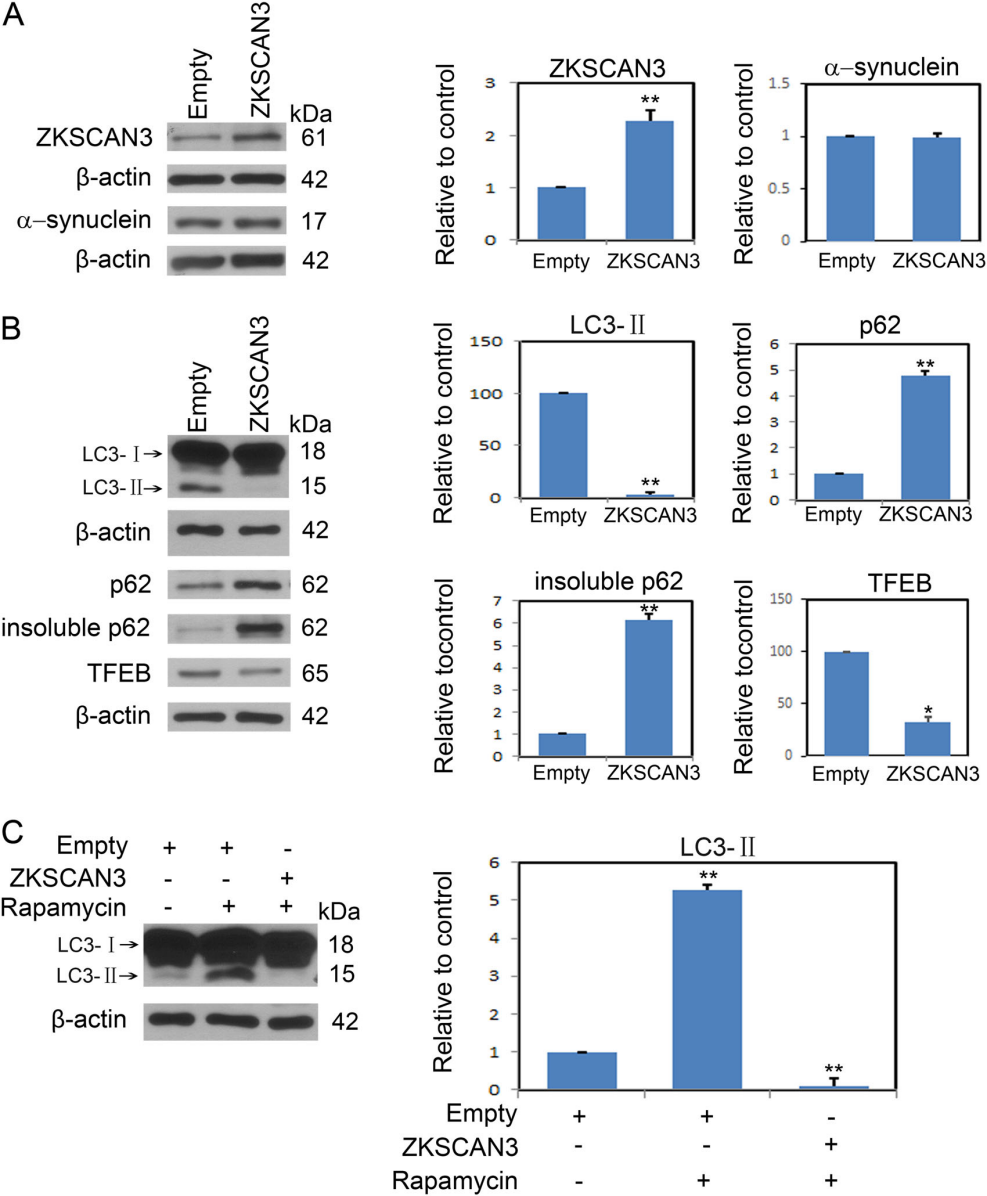
Increased ZKSCAN3 results in autophagy inhibition. **a, b** Immunoblots of lysates from midbrain dopaminergic neurons infected with either an empty or ZKSCAN3 expression AAV vector. Total protein extracts were analyzed for ZKSCAN3, α-synuclein, LC3, p62 or TFEB. The results show samples from 3 separate experiments, and quantitation of the data are shown in their corresponding right panel. **c** Midbrain dopaminergic neurons were transfected with ZKSCAN3 expression AAV vector or empty AAV vector before incubation with 100 nM rapamycin for 24 h, LC3-II levels were immunoblotted. The histogram plot right shows the densitometric quantification of LC3-II protein levels. Data points are the mean ± SEM from 3 independent experiments. *P < 0.05; **P < 0.01 by Student *t-*test

To further demonstrate that ZKSCAN3 mediates autophagy inhibition induced by A30P α-synuclein, midbrain dopaminergic neurons were transduced with a ZKSCAN3 expression AAV vector or control empty AAV vector followed by analyses of ZKSCAN3, α-synuclein, TFEB, LC3-II, and p62 levels (Fig. 4a, b). Our data clearly demonstrate that increased expression of ZKSCAN3 (Fig. 4a) independent of any other treatment results in significant decreases of both LC3-II and TFEB levels and p62 protein aggregation (Fig. 4b). Similar decreases in LC3-II levels were even found when neurons were treated with autophagy enhancer rapamycin (Fig. 4c). Collectively, these results indicate that ZKSCAN3 mediates autophagy inhibition induced by A30P α-synuclein.

### Increased A30P α-synuclein impairs autophagy without altering the activities of AMPK and mTOR

**Fig. 5:**
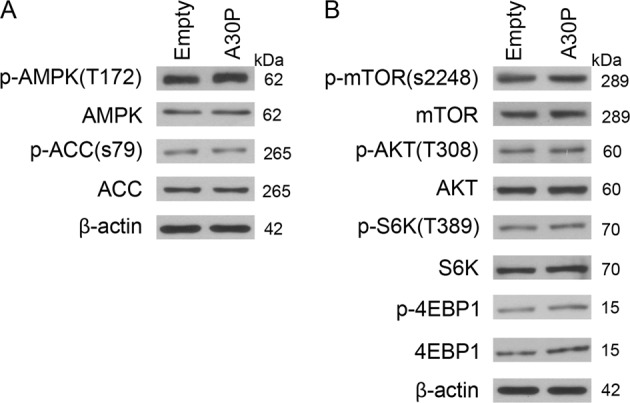
Midbrain dopaminergic neurons were infected by A30P α-synuclein AAV together with their respective control virus. The cell lysates were harvested for immunoblotting analysis as indicated above **a** and **b**. Immunoblots are representative of *n* = 3

As it was shown, the autophagy inhibition effect induced by A30P α-synuclein could not be antagonized by autophagy enhancer rapamycin (Fig. 4c). The results point out that the mechanisms underlying A30P α-synuclein-mediated autophagy impairment is not through the classical mTOR pathway. Consistent with our hypothesis, midbrain dopaminergic neurons expressing A30P α-synuclein showed no significant differences in levels of phosphorylated AMPKα at Thr172 and ACC, a substrate of AMPK, at Ser79 compared with that of control group cells (Fig. 5a). These data indicated that impaired autophagy by A30P α-synuclein was not due to a change of AMPK activity. We next investigated whether A30P α-synuclein could influence mTOR activity by detecting mTOR phosphorylation at Ser2448, the mTOR upstream mediator AKT at Thr308, the mTOR substrate p70S6K at Thr389 and phosphorylation of 4-EBP1, a downstream effector of mTORC1. The results showed that A30P α-synuclein had no effect on the alteration of mTOR activity (Fig. 5b). Therefore, these data suggested that A30P α-synuclein impaired autophagy not through altering the activities of AMPK and mTOR.

### A30P α-synuclein depresses autophagy by enhancing ZKSCAN3 nuclear translocation and activity via inhibiting JNK signaling pathway

**Fig. 6:**
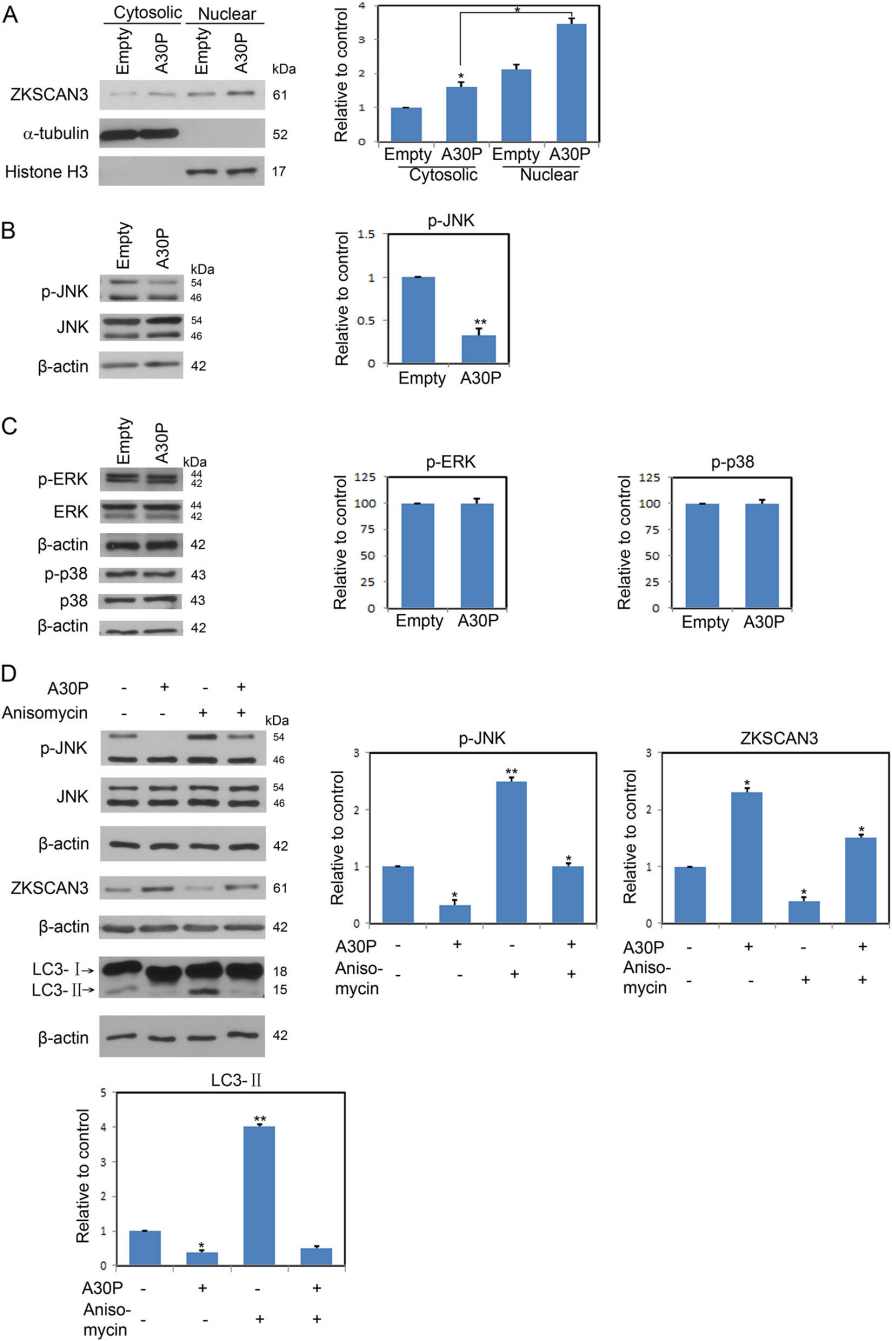
A30P α-synuclein promotes nuclear translocation of ZKSCAN3 and inhibits JNK phosphorylation. **a** Immunoblot analysis of nuclear and cytoplasmic subcellular fractions from midbrain dopaminergic neurons transduced with A30P α-synuclein AAV or empty virus. **b** A30P α-synuclein apparently inhibited p-JNK levels. **c** Midbrain dopaminergic neurons were infected by A30P α-synuclein AAV or control virus. The cell lysates were harvested for immunoblotting with the indicated antibodies. A30P α-synuclein suppressed the phosphorylation of JNK signaling pathway, while the phosphorylation of ERK and p38 was not inhibited. This inhibitory effect was rescued by anisomycin treatment **d**. The results were quantified from three independent experiments. Values are presented as mean ± SEM, ^*^P < 0.05; ^**^P < 0.01

To explore how A30P α-synuclein affects the ZKSCAN3 activity, we first examined the possibility that A30P α-synuclein invoked nuclear-cytoplasmic shuttling of ZKSCAN3. The results showed that A30P α-synuclein-induced ZKSCAN3 nuclear location in midbrain dopaminergic neurons in contrast to its cytoplasmic location under nonstressed conditions (Fig. 6a). To investigate the underlying signaling pathway of A30P α-synuclein on autophagy, we focused the attention on the JNK signaling pathway. The expression of p-JNK in the A30P α-synuclein group was significantly lower than that in the control group as reflected by the statistical analysis (Fig. 6b). The effect of A30P α-synuclein on other MAPK signaling pathways including p38 and ERK phosphorylation were also examined (Fig. 6c). Different from JNK, the phosphorylation of ERK and p38 was not affected in the neurons expressing A30P α-synuclein. The results showed that A30P α-synuclein inhibited the phosphorylation and degradation of JNK, without affecting the ERK and p38 signaling pathway. In order to further confirm that A30P α-synuclein suppressed autophagy by affecting the JNK activation, we further employed a rescue assay to validate this. As shown in Fig. 6d, p-JNK was significantly decreased in the A30P α-synuclein group compared with that in the control group. In contrast, the addition of the JNK agonist, anisomycin, apparently rescued p-JNK levels without affecting JNK protein synthesis. Although A30P α-synuclein robustly enhanced the ZKSCAN3 levels as compared to the control group, anisomycin treatment significantly reduced ZKSCAN3 levels and antagonized the effect of A30P α-synuclein on ZKSCAN3 (Fig. 6d). Anisomycin treatment increased the LC3-II levels, however, the autophagy inhibition effect in neurons expressing A30P α-synuclein could not be reverted by the administration of anisomycin (Fig. 6d). Together, these data implicated that A30P α-synuclein suppressed autophagy by inhibiting the JNK to regulate ZKSCAN3 activity.

**Fig. 7:**
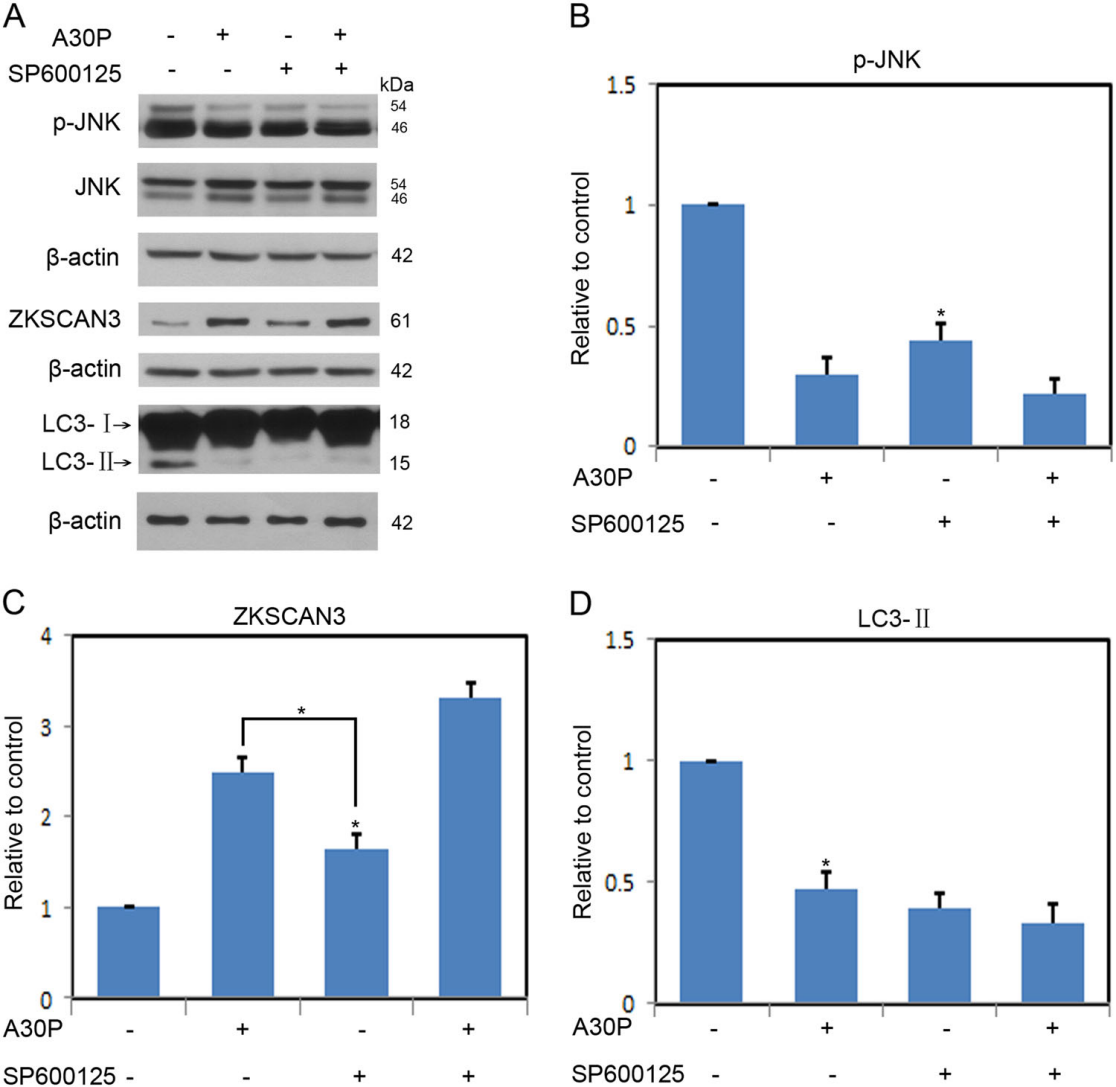
A30P α-synuclein suppresses autophagy via ZKSCAN3 in a JNK-dependent manner. **a** Midbrain dopaminergic neurons expressing A30P α-synuclein or not were treated with vehicle only, SP600125 (12.5 μM) for 24 h. Collected lysates were immunoblotted for p-JNK, JNK, ZKSCAN3, and LC3. The membranes were reprobed with a β-actin antibody as a loading control. **b**–**d** Shown is the quantification of relative levels of p-JNK, ZKSCAN3, and LC3 remaining after treatment described in **a**. Data points are the mean ± SEM from 3 independent experiments. *P < 0.05; **P < 0.01

To further determine if autophagy inhibition mediated by ZKSCAN3 in response to A30P α-synuclein overexpression was dependent on JNK inactivation in midbrain dopaminergic neuron, we used the selective JNK inhibitor SP600125. Neurons expressing A30P α-synuclein were incubated with vehicle or SP600125 (12.5 μM) for 24 h before determination of p-JNK, JNK, ZKSCAN3, and LC3 levels (Fig. 7). JNK inhibition resulted in more decreases in p-JNK in neurons expressing A30P α-synuclein than that in SP600125 treated cells alone (Fig. 7a, b). At the same time, the administration of SP600125 significantly increased the ZKSCAN3 levels in neurons expressing A30P α-synuclein compared with that in cells treated by SP600125 alone (Fig. 7a, c). Further, SP600125 significantly attenuated the decreases in the LC3-II levels in response to A30P α-synuclein overexpression (Fig. 7a, d).

## Discussion

Autophagy is known to be impaired in PD, however, the link between this dysfunction and the intracellular proteinaceous aggregates deposition has remained unclear. Accumulating evidence suggests that pathogenic A30P missense mutation in α-synuclein results in an imbalance between its production and degradation rates, leads to its accumulation, and causes severe early-onset form of PD with an autosomal dominant pattern of inheritance. A30P mutant α-synuclein inhibits CMA and causes a compensatory upregulation of macroautophagy^26^. In this study, we describe a novel mechanism in which ZKSCAN3 mediates the A30P α-synuclein-induced autophagy inhibition. Furthermore, this effect elicited with A30P α-synuclein is in a JNK signaling-dependent manner.

As a receptor and substrate of autophagy, p62 directly binds to LC3 via its LIR motif and is primarily degraded through selective autophagy^27,28^. The impaired autophagic activity results in the increase of p62, therefore, levels of p62 have been used to monitor autophagic flux^29^. Formation of Lewy bodies in the cytoplasm of midbrain dopaminergic neuron is one characteristic neuropathological hallmark of PD. These bodies consist primarily of misfolded or mutant α-synuclein and p62^30^. Mutant α-synuclein interferes with autophagic flux, the attenuation of autophagic flux leads to more α-synuclein aggregates formation and marked accumulation of p62. P62 accumulation further increases p62 expression by persistently activating Nrf2 through a positive feedback loop, which mediates protein aggregation and exacerbates autophagy dysfunction^31,32^. Moreover, due to its polymeric nature, p62 forms detergent-insoluble oligomers and bind both ubiquitinated cargoes and LC3 prior to incorporation into autophagosomes and degradation by lysosomes^33–35^. P62 aggregation is an indication of inhibited autophagic activity^36^. Thus, we examined levels of both total and detergent-insoluble p62. The results showed a significant increase in the levels of p62 in both the soluble and insoluble fractions in A30P α-synuclein-treated neurons, which indicate the reduced autophagic activity. The suppressed autophagy leads to the buildup of insoluble p62 in A30P α-synuclein-treated neurons. In addition, 3-MA is a pharmacological agent that inhibits autophagy by suppressing class III PI3K activity. The administration of 3-MA did not further increase the A30P α-synuclein-mediated p62 expression (Fig. 1e), suggesting the inhibitive effects of A30P α-synuclein may involve in autophagy flux^37^. These pronounced responses indicate the p62 accumulation and aggregation in A30P α-synuclein-treated neurons is due to inefficient autophagic clearance.

Transcriptional regulation plays a vital role in regulating autophagy. ZKSCAN3 functions in vivo as a negative regulator of autophagic flux^24^. ZKSCAN3 activation result in dysfunctional autophagy regulation, reduction of autophagosome formation and impairment of autophagosome-lysosomes fusions and cargo degradation^21^. A30P α-synuclein demonstrates increased expression of ZKSCAN3. Corresponding changes in LC3-II are further revealed in reduced responsiveness to A30P α-synuclein overexpression. In contrast, when neurons were transduced with A30P α-synuclein AAV, there was no detected change of transcription factor EB (TFEB), a master regulator associated with autophagy and lysosome biogenesis (Fig. 2). In addition, A30P α-synuclein showed no effect on LC3 cleavage. The levels of LC3-II correlate well with the number of autophagosomes present in the cytoplasm and are hence used for autophagic flux assessment. A30P α-synuclein clearly decreased the synthesis rather than enhanced degradation of LC3-II. A30P α-synuclein overexpression elicits the decreased LC3-II and the increased p62 protein levels, suggesting the repressed autophagy. Despite the results for assaying autophagic flux are solid, applying immunoblotting to assess the effect of A30P α-synuclein on autophagy only provides the one-way evidences. Visualizing autophagosomes formation via fluorescence (e.g., mRFP-GFP-LC3 vectors) may show new insights into what is happening to the impairment resulted from A30P α-synuclein. Moreover, ZKSCAN3 shRNA directly increased the downstream protein (MAP1LC3B and GABARAPL2) expression of ZKSCAN3 at the transcriptional level and significantly antagonized the autophagy inhibition effect induced by A30P α-synuclein (Fig. 3). Next, we checked the effect of ZKSCAN3 overexpression on autophagy in midbrain dopaminergic neuron. Increased ZKSCAN3 significantly reduced levels of LC3-II and TFEB and elicited p62aggregation (Fig. 4a, b). These results suggest that A30P α-synuclein compromise autophagosome formation via ZKSCAN3.

Regulation of autophagy is governed by both mTOR-dependent and -independent pathways^38^. Rapamycin is an mTOR-dependent autophagy enhancer. Inhibition of mTORC1 by rapamycin has small or no effects on TFEB activation^39^. Our studies showed that rapamycin could not reverted the ZKSCAN3 overexpression induced autophagy inhibition in midbrain dopaminergic neuron (Fig. 4c). The data indicated that A30P α-synuclein-mediated autophagy inhibition was not through mTOR-dependent autophagy pathway. Since neither mTOR nor AMPK activity can be modulated in neurons expressing A30P α-synuclein (Fig. 5), we focused our attention to the c-Jun N-terminal kinase (JNK) signal transduction pathway. JNK modulates autophagy at multiple regulatory levels. JNK can promote autophagy by stimulating LC3 gene expression and controlling LC3 lipid conjugation^40^. JNK also phosphorylates ZKSCAN3, facilitate its nuclear export and leads to de-repression of autophagy gene expression^25^. Here we found that A30P α-synuclein overexpression suppressed JNK activity as suggested by decreased p-JNK levels (Fig. 6b). In contrast, A30P α-synuclein exhibited no inhibitory effect on both ERK and p38 activity in midbrain dopaminergic neuron (Fig. 6c). In addition, the addition of JNK agonist anisomycin rescued A30P α-synuclein-induced JNK inactivation and reversed its inhibitory effect on autophagy flux in midbrain dopaminergic neuron (Fig. 6d). Furthermore, the inhibition of JNK with SP600125 increased ZKSCAN3 protein levels and further decreased LC3-II levels compared with that in neurons expressing A30P α-synuclein alone group (Fig. 7). These data demonstrate that A30P α-synuclein-induced autophagy inhibition is dependent on increased ZKSCAN3 activity and works through inactivation of JNK.

Together, these studies provide clear evidence that the familial A30P mutant a-synuclein impedes midbrain dopaminergic neuron autophagy flux via activation of autophagy transcriptional repressor ZKSCAN3 in a JNK-dependent manner. This findings explore a novel mechanism of A30P α-synuclein in regulating autophagy and may present a new therapeutic target for PD.

## Materials and methods

### Antibodies and chemicals

The antibodies used in the study were as follows: anti-ZKSCAN3 and anti-Atg4B from Abcam. Anti-human α-synuclein from Invitrogen. Anti-α-synuclein from BD. Anti-LC3B, anti-SQSTM1/p62, anti-phospho-4E-BP1 S65, anti-4E-BP1, anti-phospho-ACC S79, anti-ACC, anti-phospho-AMPKα T172, and anti-AMPKα, anti-phospho-p70S6K (Thr389), anti-p70S6K, anti-phospho-mTOR (Ser2448), anti-mTOR, anti-phospho-AKT (Thr308) and anti-TFEB from Cell Signaling Technology. Anti-phospho-JNK (Thr183/Tyr185), anti-JNK, anti-phospho-ERK1/2 (Tyr204), anti-ERK1/2, anti-phospho-p38 (Tyr182), anti-p38, and anti-AKT from Santa Cruz Biotechnology. The other antibodies used in the study were anti-β-actin (Millipore), and anti-α-tubulin (Pierce Biotechnology). SP600125 and rapamycin was purchased from Cell Signaling Technology; 3-methyladenine (3-MA), cycloheximide (CHX), anisomycin and chloroquine (CQ) from Sigma-Aldrich. Bafilomycin A1 was purchased from LC Laboratories.

### Plasmid preparation

Human α-synuclein (α-syn, WT), A30P α-synuclein cDNAs or ZKSCAN3 ORF were inserted into the pAAV-mPGK-MCS backbone, modified from the serotype 2 pAAV-CMV-MCS using standard cloning procedures. EGFP was cloned into the pAAV-mPGK-MCS backbone using standard cloning procedures. Recombinant pseudotyped rAAV2/6 were produced, purified and titrated as described before^41^. The titers measured for AAV-pgk-α-syn-A30P and AAV-pgk-GFP used were 1 × 10^11^ TU/ml and 2.6 × 10^10^ TU/ml, respectively. rAAV ZKSCAN3-sh and rAAV ZKSCAN3-scr (scramble) were generated by ligating annealed oligonucleotides targeting endogenous ZKSCAN3 (5’-TATCGTGCCACCTGAGAGA-3’) or a control sequence (5’-CAGTCGCGTTTGCGACTGG-3’) into the pA2SGW-Synapsin-GFP-U6-siRNA rAAV vector. High titer and purity rAAV vectors were generated by cotransfection of HEK293 cells with the rAAV backbone plasmid and the helper AAV6 plasmid, using the calcium-phosphate method, as previously described. The final titer of the used rAAVs was 3.4 × 10^8^ TU/μl, 3.2 × 10^8^ TU/μl for rAAV6 expressing ZKSCAN3-sh and ZKSCAN3-scr, respectively.

### Cell culture, drug treatment, and transfections

All animal experiments were performed in accordance with protocols approved by the Southeast University Animal Care and Use Committee. Ventral mesencephalic (VM) neurons were dissected from E14 Sprague-Dawley rat embryos. In brief, VM tissue was incubated in 0.1% trypsin and 0.05% DNase in Dulbecco’s modified Eagle medium (DMEM) for 20 min at 37 °C. Cells were washed four times with 0.05% DNase in DMEM and triturated until a single cell suspension was reached. Neurons were plated at a density of 10^4^ cells/cm^2^ on 0.01% poly-L-lysine coated 6-well plates. Neurons were kept in DMEM with 10% FBS for 3 h, then media was changed to Neurobasal media supplemented with 2% B27, 0.5 mM GlutaMax and 1% antibiotic-antimycotic. For the A30P mutant α-synuclein expression, the days in vitro (DIV) 4 neurons were transduced with A30P α-syn-AAV at a multiplicity of infection of 33 in a half volume of fresh growth medium. Following 90 min of incubation, the remaining half volume of fresh growth medium was added, and the cells were incubated overnight. The next day, the medium was replaced with fresh medium and incubated further before treatment. For drug treatment, neurons were used at DIV 7. Cells were treated with 10 nM bafilomycin A1 for 12 h or 10 mM 3-MA, 10 μM CQ, 100 nM rapamycin, 0.25 μM anisomycin, 12.5 μM SP600125 for 24 h in growth medium prior to analysis. Cycloheximide was used at 10 μg/ml for the indicated periods of time. For the ZKSCAN3 knockdown experiments, the neurons were infected with AAV on DIV 4 after the half-medium change by adding the virus particles to the medium at a 1:100 (viral suspension:neurobasal medium) ratio for 12 h. The transduction media was then fully replaced with 50% conditioned and 50% fresh neurobasal medium (with 0.5 mM glutamine, 2% B27). Neurons were then treated as indicated on DIV 9 and collected on DIV 10. For the ZKSCAN3 overexpression studies, neurons were transduced on DIV 2 and again on DIV 4 and collected on DIV 8 for analysis.

### Measurement of cell viability

Midbrain dopaminergic neurons were plated on 24-well plates and on DIV7 cells were treated as indicated and viability measured after 24 h. Neuronal viability was assayed by measuring the change in fluorescence intensity following the cellular reduction of resazurin to resorufin. All experiments were performed in cell culture medium at 37 °C. Cell viability was assessed incubating cells in 10% resazurin for 45 min at 37 °C, then measuring fluorescence at 540/590 nm (excitation/emission). This fluorescence was expressed as a percentage of that in control cells (which were in cell culture medium only), after subtraction of background fluorescence.

### Quantitative real-time PCR and luciferase assay

Total mRNA from VM cultures was extracted using the RNeasy Mini Kit (Qiagen) and was reverse transcribed to cDNA using SuperScript III First-strand synthesis Supermix from Life Technologies according to the manufacturer’s instructions. Quantitative real-time PCR was carried out using SYBR Green mix (Life Technologies) and RT-PCR thermocycler (Biorad). Fold difference was calculated by the comparative C_T_(2^−∆∆C^T) method against GAPDH. Primers used are as below: SQSTM1/p62 Forward: 5′-AAGAACGTTGGGGAGAGTGT-3′, SQSTM1/p62 Reverse: 5′-CTGTGCTGGAACTCTCTGGA-3′ and GAPDH Forward: 5′-GAGTC AACGGATTTGGTCGT-3′, GAPDH Reverse: 5′-TGGAAGATGGTGATGGGATT-3′. For luciferase assay, *MAP1LC3B and GABARAPL2* promoter-reporter constructs in pGL3 vector were generated as described previously^21,42,43^. These luciferase reporter constructs along with *Renilla* luciferase expression vector were transiently transfected in midbrain dopaminergic neurons expressing A30P α-synuclein transfected with either scramble (SCR) or ZKSCAN3-shRNA virus. After 48 h transfection, luciferase assays were performed using dual luciferase reporter assay kit with Renilla luciferase activity as control (Promega, USA). Each transfection was performed in triplicate, each consisting of three wells per transfection.

### Subcellular fractionation

Cell pellets were resuspended in lysis buffer (10 mM Hepes pH 7.9, 10 mM KCl, 0.1 mM EDTA and 0.4% Nonidet P40) with inhibitors by pipetting and kept in ice for 30 min. After 1 min of spin at full speed, the supernatant was collected as cytosolic fraction. The pellet was washed twice with lysis buffer and resuspended with nuclear buffer (20 mM Hepes pH 7.9, 0.4 M NaCl and 1 mM EDTA) containing phosphatases and proteases inhibitors. After 15 min of vigorous shaking on an Eppendorf shaker, the pellet was spun down at full speed for 10 min. The supernatant was used as the nuclear fraction.

### Western immunoblot analysis

All immunoblotting procedures have been described elsewhere. Briefly, cells were rinsed in ice-cold phosphate-buffered saline and collected in lysis buffer, containing 0.5% NP-40, 150 mM NaCl, 10 mM Tris-Cl (pH 7.4), 1 mM EGTA, 1 mM EDTA, 1 mM phenylmethylsulphonyl fluoride, and 10 μg/mL each of aprotinin, leupeptin, and pepstatin. Samples were sonicated on ice and centrifuged at 13,200×*g* for 20 min, and the supernatant and the detergent-insoluble pellets were separated for further analysis. The detergent-insoluble pellets were solubilized in 1 × SDS-PAGE loading buffer and sonicated briefly. Samples containing equal amounts of proteins were boiled in Laemmli denaturing buffer, size-fractionated by SDS-PAGE, and transferred onto a nitrocellulose membrane (Bio-Rad). Blots were blocked in 5% nonfat dry milk in TBST (20 mM Tris-HCl, pH 7.6, 137 mM NaCl, 0.05% Tween 20) for 1 h at room temperature. The membranes were incubated overnight with the primary antibody (diluted in TBS-Tween), washed 3 times with TBST and incubated with HRP-conjugated secondary antibody for 1 h at room temperature. The membranes were then rinsed 3 times for 30 min with TBST, followed by 4 quick rinses with distilled water and developed with enhanced chemiluminescence (ECL, Bio-Rad). Protein expression levels were quantified by densitometric analysis of immunoreactivity, using the ImageJ 1.48 v software. Data were evaluated for significance by Student *t*-test and one-way ANOVA.

### Statistical analysis

Data plotting and statistical analysis were performed using statistical software (Microsoft Excel 2013 and SPSS20.0). All data were expressed as mean ± SEM. Differences between groups were analyzed by Student unpaired *t*-test for 2 groups or one-way ANOVA analysis with Tukey’s post-hoc test, and with a *P* < 0.05 considered statistically significant.

## Supplementary information

supplementary figure legend

supplementary figure 1

supplementary figure 2
