# Supplementary material for: A30P mutant α-synuclein impairs autophagic flux by inactivating JNK signaling to enhance ZKSCAN3 activity in midbrain dopaminergic neurons
Source: Cell Death Dis. 2019 Feb 12;10(2):133. doi: 10.1038/s41419-019-1364-0 (PMC6372582; doi:10.1038/s41419-019-1364-0)
Supplement: Supplementary file 1 — supplementary figure legend [file 41419_2019_1364_MOESM1_ESM.docx]

**Supplementary Figure legend**

**Fig. S1** 3-methyladenine (3-MA) increases the levels of p62 in a dose-dependant manner. **a** Midbrain dopaminergic neurons were treated by 3-MA (0-15mM) for 24 h. Cell lysates were subjected to immunoblot analysis using antibodies to p62 or β-actin. The levels of p62 were quantified by densitometry and normalized with β-actin. **b** Midbrain dopaminergic neurons were treated with 3-MA (0-15mM) for 24 h and cell viability was determined with the resazurin assay. Data are represented as the mean ± SEM from three independent experiments. ^*^*P*<0.05.

**Fig. S2** ZKSCAN3 shRNA has no effect on the autophagy inhibition induced by WT α-synuclein, as well as it counteracts the impaired cell viability triggered by A30P α-synuclein. **a** Immunoblots of cell lysates from midbrain dopaminergic neurons transfected with empty or WT α-synuclein vectors. Antibodies to human α-synuclein or α-synuclein were used to detect only the α-synuclein expressed by the AAV or the total cellular α-synuclein levels. **b** Representative immunoblots of cell lysates from midbrain dopaminergic neurons expressing WT α-synuclein were infected with either scramble (SCR) or ZKSCAN3 shRNA virus. Total protein extracts were immunoblotted for LC3, ZKSCAN3, TFEB or p62. **c-f** shown is the densitometric quantification of corresponding protein levels described in **b. g** Cell viability was assessed by the resazurin assay. Midbrain dopaminergic neurons were transfected with the given vectors. Error bars represent means ± SEM from three independent experiments. Statistics: one-way ANOVA with Tukey’s post-hoc correction followed by Student *t* test, ^*^*P*<0.05; ^**^*P*<0.01.
