# Supplementary figures and images for: A30P mutant α-synuclein impairs autophagic flux by inactivating JNK signaling to enhance ZKSCAN3 activity in midbrain dopaminergic neurons
Source: Cell Death Dis. 2019 Feb 12;10(2):133. doi: 10.1038/s41419-019-1364-0 (PMC6372582; doi:10.1038/s41419-019-1364-0)

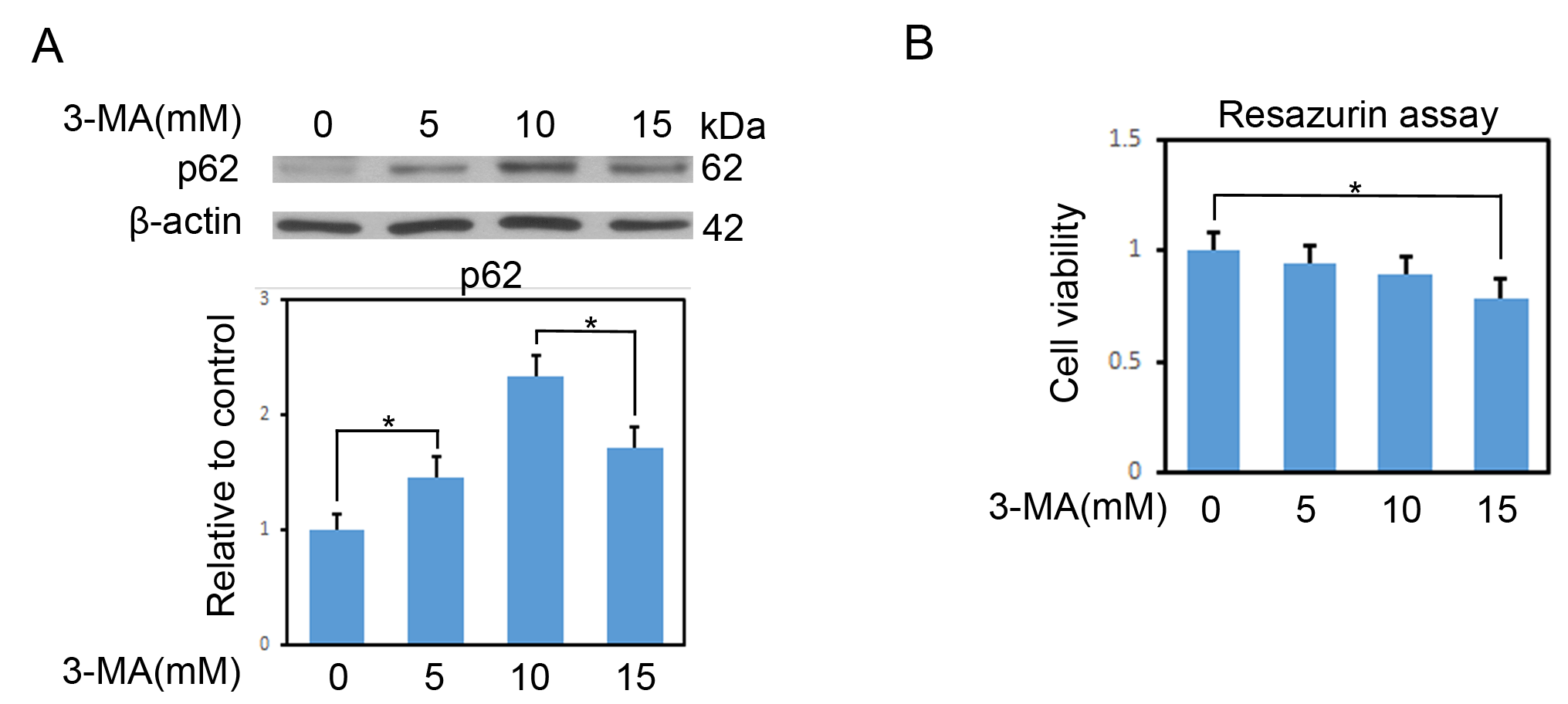

Supplement: Supplementary file 2 — supplementary figure 1 [file 41419_2019_1364_MOESM2_ESM.tif]

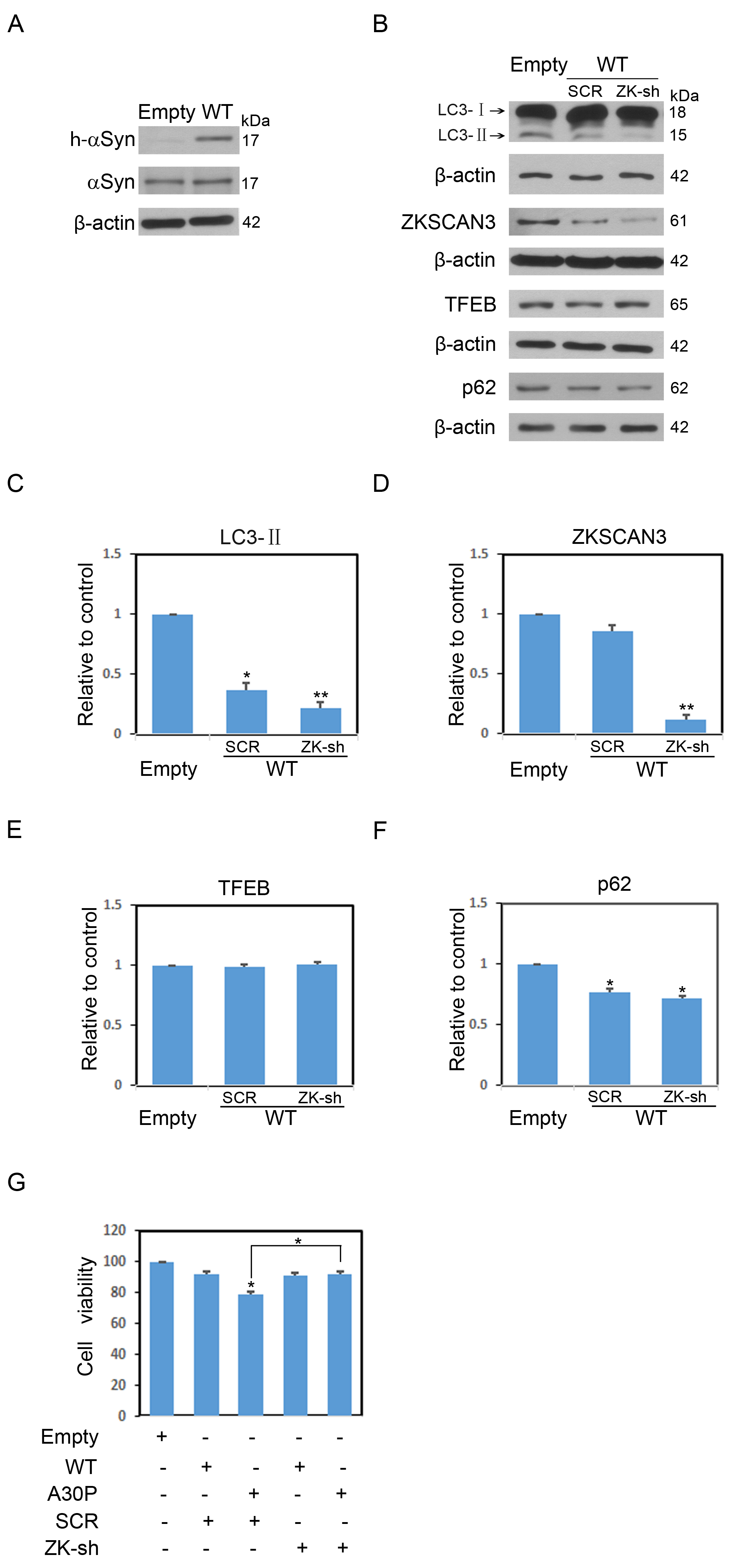

Supplement: Supplementary file 3 — supplementary figure 2 [file 41419_2019_1364_MOESM3_ESM.tif]
